# Orexin prevents depressive-like behavior by promoting stress resilience

**DOI:** 10.1038/s41380-018-0127-0

**Published:** 2018-08-07

**Authors:** Miao-Jin Ji, Xiao-Yang Zhang, Zi Chen, Jian-Jun Wang, Jing-Ning Zhu

**Affiliations:** 1 State Key Laboratory of Pharmaceutical Biotechnology and Department of Physiology, School of Life Sciences, Nanjing University, 163 Xianlin Avenue, Nanjing, 210023 China; 2 Institute for Brain Sciences, Nanjing University, 163 Xianlin Avenue, Nanjing, 210023 China

**Keywords:** Neuroscience, Depression

## Abstract

Hypothalamic neuropeptide orexin has been implicated in the pathophysiology of psychiatric disorders and accumulating clinical evidence indicates a potential link between orexin and depression. However, the exact role of orexin in depression, particularly the underlying neural substrates and mechanisms, remains unknown. In this study, we reveal a direct projection from the hypothalamic orexinergic neurons to the ventral pallidum (VP), a structure that receives an increasing attention for its critical position in rewarding processing, stress responses, and depression. We find that orexin directly excites GABAergic VP neurons and prevents depressive-like behaviors in rats. Two orexin receptors, OX1R and OX2R, and their downstream Na^+^–Ca^2+^ exchanger and L-type Ca^2+^ channel co-mediate the effect of orexin. Furthermore, pharmacological blockade or genetic knockdown of orexin receptors in VP increases depressive-like behaviors in forced swim test and sucrose preference test. Intriguingly, blockage of orexinergic inputs in VP has no impact on social proximity in social interaction test between novel partners, but remarkably strengthens social avoidance under an acute psychosocial stress triggered by social rank. Notably, a significantly increased orexin level in VP is accompanied by an increase in serum corticosterone in animals exposed to acute stresses, including forced swimming, food/water deprivation and social rank stress, rather than non-stress situations. These results suggest that endogenous orexinergic modulation on VP is especially critical for protecting against depressive reactions to stressful events. The findings define an indispensable role for the central orexinergic system in preventing depression by promoting stress resilience.

## Introduction

Depression is a prevalent and life-threatening psychiatric disorder and one of the leading causes of disease burden worldwide [1, 2]. Its core symptoms include feelings of despair, inability of experience pleasure (anhedonia) as well as social avoidance [3, 4]. Although recent years have witnessed great progresses in our understanding of the neural and molecular mechanisms of depression, the neural circuits underlying depression and their exact functional roles remain enigmatic. The ventral pallidum (VP), a long neglected structure in the basal ganglia, attracts increasing attention recently. Accumulating evidence indicates that VP is involved in not only motivational salience and addiction but also emotion regulation [5, 6]. Furthermore, patients with lesions of bilateral VPs show anhedonia to sucrose and social partners [7, 8]. In addition, a smaller baseline VP volume has been reported in depressive patients [9]. Therefore, VP is implicated as a core component of the mesocorticolimbic circuits for reward [10, 11] and depression [12]. Yet little is known about the endogenous factors modulating VP activity.

Notably, depressive patients exhibit a reduced level of orexin in cerebrospinal fluid [13]. Orexin is a neuropeptide restrictedly synthesized in the hypothalamus, but extensively modulates the whole brain activity and regulates a variety of complex behaviors, such as feeding, sleep/wakefulness, reward, and emotion [14–18]. It has been well known that the absence of orexin results in narcolepsy-cataplexy [19, 20], an excessive daytime sleepiness companied by a sudden loss of muscle tone often triggered by strong emotions. Intriguingly, patients with narcolepsy-cataplexy also manifest moderate to severe depressive symptoms [21, 22]. These clinical clues indicate an emerging role of the central orexinergic system in the pathophysiology of depression and prevention of depression. Therefore, in this study, we dissect the neural substrates responsible for preventing function of the central orexinergic system on depression, particularly the pathways through the VP. We demonstrate that VP receives direct innervation from the hypothalamic orexinergic neurons, and orexin directly excites GABAergic VP neurons via two orexin receptors, OX1R and OX2R. Given the findings that blockage of orexinergic inputs in VP by knocking down orexin receptors induces depressive-like behaviors in paradigms with, rather than without, acute stress, we attribute the protective role of the central orexinergic system against depression, partly but substantially, to promotion of stress resilience via its direct modulation on VP activity.

## Materials and methods

See Supplementary Information for details, methods are briefly described as follows.

### Animals

Adult male Sprague-Dawley rats (8 weeks, 200–250 g) were housed under standard animal housing conditions except for a specific description. All experimental procedures were approved by the Experimental Animal Care and Use Committee of Nanjing University.

### Retrograde tracings

The retrograde tracer Fluoro-Gold (FG) was micro-electrophoresed in VP (A 0.0 to −0.3, L 2.5, and H 7.6) following our previous reports [23, 24]. The immunohistochemical experiment was performed 2 weeks later to determine the injection site and location of retrogradely labeled cells.

### Immunohistochemistry

Sections with FG were immunolabelled with primary antibodies against orexin-A (1:200; R&D, Minneapolis, MN; Cat# MAB763, RRID:AB_2117627) and FG (1:2000; Millipore, Boston, MA; Cat# AB153, RRID:AB_90738) as we described previously [23, 24]. Primary antibodies to OX1R (1:100; Abcam; Cat# ab68718, RRID: AB_1269637) and OX2R (1:100; Everest, Waltham, MA; Cat# EB08124, RRID:AB_2117786) were used for detecting the expression of two orexin receptors in VP. For immunohistochemical identification of the GABAergic VP neurons recorded in the voltage and current clamp, brain slices containing the recorded neurons filled with biocytin were fixed, dehydrated, resectioned, and then incubated with primary antibodies against GABA (1:1000; Sigma, St. Louis, MO; Cat# A0310, RRID: AB_476667).

### Stereotactic microinjection and lentiviruses

Rats were submitted to stereotaxic surgery and bilateral implantation of guide cannula 2 mm above VPs (A 0.0 to −0.3, L 2.5, and H 7.6) for microinjection of saline, orexin-A (one of the endogenous orexin peptides; 1 μM, 0.5 μl/lateral), or TCS1102 (a potent dual orexin receptor antagonist; 20 nM, 0.5 μl/lateral). shRNA lentiviruses targeting OX1R and OX2R mRNAs (LV-shOX1R-eGFP and LV-shOX2R-eGFP) were produced by the lentiviral vector GV248 (pFU-GW-007-hU6-Ubiquitin-EGFP-IRES-puromycin; GeneChem, Shanghai, China) and stereotaxically microinjected (1 × 10^9^ TU/ml, 1 μl/lateral) into the bilateral VPs for knockdown of orexin receptors. The non-targeting shRNA lentiviral vector (LV-CON-eGFP) was used as control. After 2 weeks, the orexin receptor mRNA and protein levels in the VP were assessed by quantitative real-time RT-PCR and western blot [24–26].

### Behavioral assessments

Depressive-like behaviors were assessed by forced swim test (FST), sucrose preference test (SPT), and social interaction test (SIT) using Clever TopScan [27]. Social interactions between novel partners were measured as social proximity. The tested rat was placed in the center of a plastic open field apparatus (50 × 50 cm) simultaneously with the other novel partner, which was put in an iron cage (16 × 22 cm) in one corner of the arena. Time of head approaching and staying close to the cage (≤5 cm) and the times of forepaws climbing the cage in 10 min were recorded and calculated to reflect social proximity. Interactions of the subordinate with the dominator ranked by a tube test for social dominance [28, 29] were measured as social avoidance under an acute psychosocial stress. Open field test was applied to examine general locomotor activity.

### Whole-cell patch clamp recordings

Whole-cell patch clamp recordings in brain slices were applied to assess the effect of orexin on VP neurons and the underlying receptor and ionic mechanisms as previously reported [23, 25, 26, 30]. Briefly, recordings of whole-cell currents were low-pass filtered at 2 kHz and digitized at 10 kHz and recordings of membrane potentials were low-pass filtered at 5 kHz and digitized at 20 kHz.

### Enzyme-linked immunosorbent assay (ELISA)

Serum corticosterone level and orexin concentration in VP were quantified by ELISA (corticosterone kit and orexin-A kit), following the manufacturer’s instructions. After averaging the results of duplicate wells, corticosterone value of each sample was calculated as ng/ml serum and orexin value as pg/mg wet tissue.

### Data analysis

All data were analyzed with SPSS 17.0 and presented as median (horizontal bar) with 25th–75th (box) and 10th–90th (whiskers) percentiles. Two-tailed unpaired and paired *t* test, one-way, two-way, and repeated measures two-way analysis of variance (ANOVA), and post hoc Bonferroni-corrected *t* test was employed for statistical analysis. *P*-values of <0.05 were considered to be significant. The exact sample size, the statistical test used, and the results of the tests for each experiment were included in results and figure legends.

## Results

### Pharmacological blockade or knockdown of orexin receptors in VP induces depression-like behaviors

**Fig. 1:**
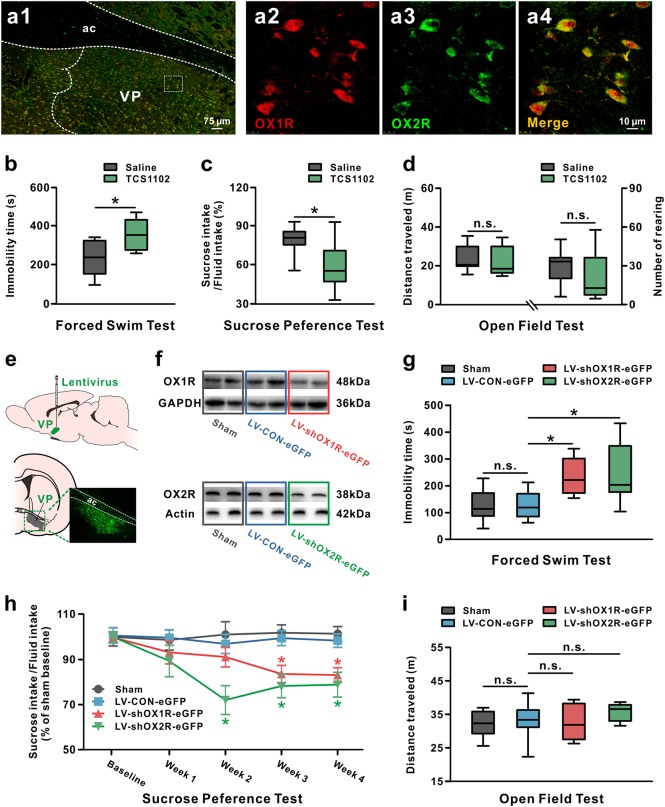
Pharmacological blockage or lentiviral-mediated knockdown of orexin receptors in VP led to depression-like behaviors. **a** Double immunostaining results showed that two orexin receptors, OX1R (red) and OX2R (green), were co-localized in all of the stained VP neurons. **b**–**d** Microinjection of TCS1102, a potent dual orexin receptor antagonist, into VP increased immobility time in the forced swim test (*n* = 11–12) (**b**) and decreased sucrose preference in the sucrose preference test (*n* = 11) (**c**), but did not influence distance traveled and number of rearing in the open field test (*n* = 11–12) (**d**). In the forced swim test and open field test, *n* = 11 for the saline group, *n* = 12 for the TCS1102 group. **e** A coronal brain section showing the microinjection site of lentivirus in VP. **f** Identification of the downregulation efficiency of OX1R and OX2R protein by western blot. **g–i** Effects of downregulation of OX1R or OX2R in VP on behaviors of rats in the forced swim test (*n* = 9–11) (**g**), sucrose preference test (*n* = 10) (**h**), and open field test (*n* = 9–11) (**i**). In the forced swim test and open field test, *n* = 9 for the sham group, *n* = 10 for the LV-Control-eGFP and LV-shOX1R-eGFP group, *n* = 11 for the LV-shOX2R-eGFP group. Data are represented as median (horizontal bar) with 25th–75th (box) and 10th–90th (whiskers) percentiles; n.s. indicates not significant and *P < 0.05, by two-tailed unpaired *t*-test (**b**–**d**), one-way ANOVA (**g**, **i**), or repeated measures two-way ANOVA (**h**) followed by Bonferroni-corrected *t* test. ac anterior commissure, VP ventral pallidum

Two orexin receptor subtypes, OX1R and OX2R [31, 32], are known to mediate orexin functions. In the present study, we first identify the expression of orexin receptors in VP in rats. Double immunostaining results showed that both OX1R and OX2R were co-localized in VP neurons (Fig. 1a1–a4).

Next, we blocked orexin receptors in VP to determine the role of endogenous orexinergic system in the depressive-like behaviors in FST and SPT. Both of these tests are classical depressive paradigms related to two core symptom domains, despair and anhedonia, in depression, respectively [33]. Pharmacological blockade of both OX1R and OX2R by microinjection of TCS1102, a potent dual orexin receptor antagonist, into bilateral VPs, led to a significant increase in immobility time in the FST (*P* < 0.05; Fig. 1b) and a remarkable decline in sucrose intake in the SPT (*P* < 0.05; Fig. 1c), but did not influence the total distance traveled (*P* = 0.612; Fig. 1d) and number of rearing in the open field test (*P* = 0.261; Fig. 1d).

To further assess the contribution of each subtype of orexin receptors to the orexin-induced reduction of depressive-like behaviors, we generated lentiviral vectors carrying shRNA targeting OX1R (LV-shOX1R-eGFP) or OX2R mRNAs (LV-shOX2R-eGFP) to separately knockdown the expression of OX1R and OX2R in VP. The extent of lentivirus infection in VP was identified by detecting a fluorescent tag eGFP coexpressed with shRNA (Fig. 1e), and the effectiveness of knockdown was examined by qPCR (Supplementary Figure 1a) and western blot (Fig. 1f and Supplementary Figure 1b). OX1R/OX2R shRNA lentivirus significantly decreased the level of OX1R and OX2R mRNAs to 42.04 ± 0.02% and 65.36 ± 0.01% (*P* < 0.001, respectively; Supplementary Figure 1a), and accordingly reduced the level of OX1R and OX2R proteins to 46.20 ± 0.04% and 32.88 ± 0.04% (*P* < 0.01 and *P* < 0.001; Supplementary Figure 1b), respectively. In line with the above pharmacological results, we found that either OX1R or OX2R knockdown in VP significantly prolonged the immobility time of rats in the FST (*P* < 0.05, respectively; Fig. 1g), and maximally suppressed the sucrose preference at 3 (*P* < 0.05, respectively) and 2 weeks (*P* < 0.05, respectively) after lentivirus administration, followed by a low plateau (Fig. 1h). However, knockdown of OX1R or OX2R had no effect on general locomotor activity in the open field (*P* = 0.340; Fig. 1i). These behavioral results from pharmacological and molecular manipulations collectively suggest that blockage of endogenous orexinergic inputs in VP results in depressive-like behaviors, and OX1R and OX2R may have the homogeneous contribution to the protective effect of orexin on depression.

### Orexin depolarizes GABAergic VP neurons and prevents depressive-like behaviors

**Fig. 2:**
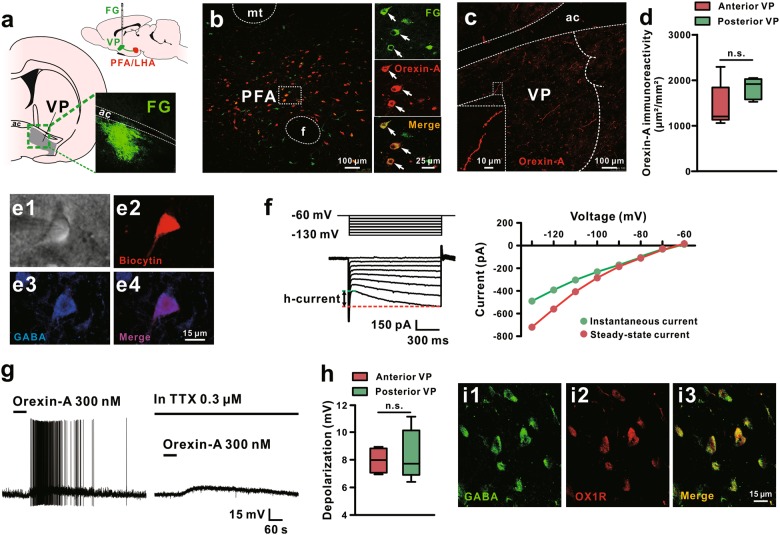
Orexinergic neurons in the hypothalamus directly project to VP and orexin depolarizes GABAergic VP neurons. **a** Diagram and a coronal brain section showing the identification of PFA/LHA-VP orexinergic projections with injections of FG into the VP. **b** Double immunoreactivity of orexin-A (red) and FG (green) in the same PFA/LHA neurons. **c** Orexinergic fibers in the VP and their varicosities. **d** Surface area of immunoreactivity for orexinergic fibers in the anterior and posterior VP of rats (*n* = 5). **e**, **f** Morphological, immunohistochemical, and electrophysiological identifications of the recorded GABAergic neurons in the VP. Based on infrared differential interference contrast images, VP neurons with diameters around 15 μm were patched as candidate GABAergic neurons (**e1**). Immunochemistry was used to identify GABAergic VP neurons by immunostaining the recorded biocytin-filled neurons with GABA (**e2-e4**). A series of 1 s hyperpolarizing voltage steps (ranging from −60 to −130 mV in 10 mV steps) were employed to observe inwardly rectifying *h*-currents, a feature of GABAergic VP neurons, in recorded neurons (**f**). **g** Orexin-A depolarized the recorded VP neurons and brought up the neurons firing. The orexin-induced depolarization on VP neurons did not blocked by TTX, suggesting a direct postsynaptic effect of orexin. **h** Group data of the tested VP neurons (*n* = 5 for anterior and posterior VP, respectively). **i** Double immunoreactivity of GABA (green) and OX1R (red) in the same VP neurons. mt mammillothalamic tract, aca anterior commissure, ant, PFA perifornical nucleus, VP ventral pallidum. Data are represented as median (horizontal bar) with 25th–75th (box) and 10th–90th (whiskers) percentiles; n.s. indicates not significant, by two tailed unpaired *t*-test (**d**, **h**)

To investigate whether orexinergic neurons directly project to VP, we micro-electrophoresed FG into VP (Fig. 2a), and found that a number of orexin-immunopositive neurons in the perifornical/lateral hypothalamic area (PFA/LHA), the origin of the central orexinergic system, were retrogradely labeled with FG (Fig. 2b). Moreover, the orexin-immunoreactive fibers with bead-like varicosities were scattered throughout the VP (Fig. 2c, d). Although the density of orexinergic fibers was slightly higher in the posterior VP, there is no statistically significant difference between anterior and posterior VP (*P* = 0.145; Fig. 2d). These results indicate a direct projection of PFA/LHA orexinergic neurons to VP and a possible modulation of orexin released by the orexinergic terminals on VP neuronal activities.

Since GABAergic neurons are the majority and principle neurons in VP [34, 35], we used whole-cell patch clamp recordings on brain slices to assess the effect of orexin on VP GABAergic neurons. We filled all recorded neurons with biocytin after recordings and then immunostained them with GABA. The recorded GABAergic VP neurons co-labeled by biocytin and GABA in this study had small sized (somata diameters around 15 μm, Fig. 2e1–e4), and characteristically exhibited a prominent *h*-current, a slowly hyperpolarization activated inwardly rectifying current (Fig. 2f). These morphological and electrophysiological properties are consistent with previous reports [35, 36]. We found that orexin-A evoked a strong depolarization on GABAergic VP neurons (10/11, 90.9%), which were silent at rest, and sufficiently brought up the neurons firing (Fig. 2g, left panel). In addition, orexin induced a depolarization in the presence of TTX (Fig. 2g, right panel) and there is no significant difference between the amplitude of the orexin-induced depolarization on GABAergic neurons of anterior and posterior VP (*P* = 0.668; Fig. 2h), suggesting a direct postsynaptic excitatory effect of orexin on GABAergic VP neurons. Consistently, double immunostaining results showed that OX1R is expressed on the GABA-positive neurons in VP (Fig. 2i1–i3).

**Fig. 3:**
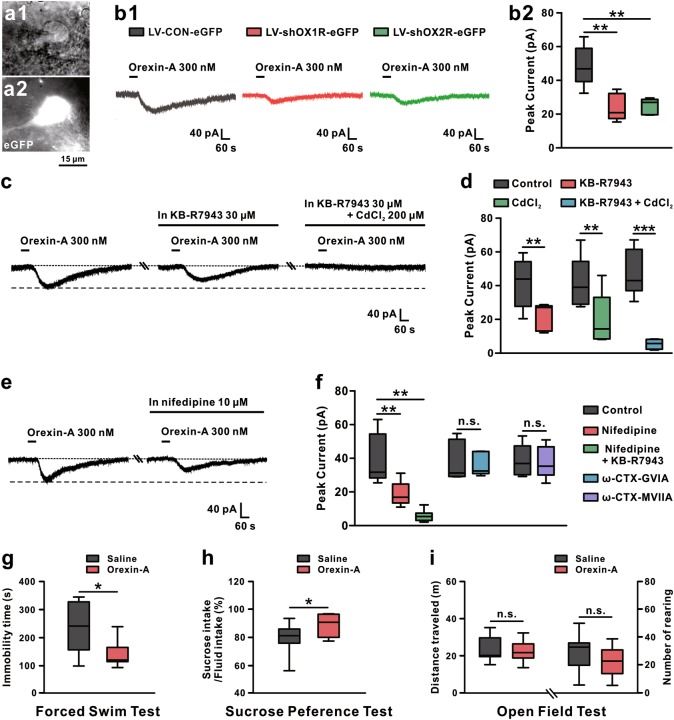
The receptor and ionic mechanisms and behavioral effects of orexin-induced excitation on VP neurons. **a1**–**a2** Based on infrared differential interference contrast and fluorescence images, lentivirus-infected eGFP-positive VP neurons with diameters around 15 μm were recorded. **b1** Knockdown of OX1R (*n* = 5) or OX2R (*n* = 6) significantly reduced the orexin-A-induced inward current (*n* = 6) on VP neurons. **b2** Group data of the tested VP neurons. **c** KB-R7943, a selective blocker of NCX (*n* = 5), partly blocked the orexin-A-elicited inward current, and combined application of KB-R7943 and CdCl_2_ totally blocked the current (*n* = 6). **d** Group data of the tested VP neurons. **e** Nifedipine, a selective L-type Ca^2+^ channel blocker (*n* = 6), partially inhibited the orexin-A-induced inward current. **f** Group data of the tested VP neurons. **g**–**i** Microinjection of orexin-A into VP decreased immobility time in forced swim test (*n* = 11) (**g**) and increased sucrose preference (*n* = 11) (**h**), but did not influence distance traveled (*n* = 11) and number of rearing (*n* = 11) in the open field (**i**). Control in **b**, **d**, **f** refers to orexin-A treatment. Data are represented as median (horizontal bar) with 25th–75th (box) and 10th–90th (whiskers) percentiles; n.s. indicates not significant and *P < 0.05, **P < 0.01, ***P < 0.001, by one-way ANOVA (**b**) followed by Bonferroni-corrected *t* test, two tailed paired (**d**, **f**) or unpaired *t*-test (**g**–**i**)

Next, we determined the receptor and ionic mechanisms underlying the orexin-induced excitation on GABAergic VP neurons. Selective OX1R antagonist SB334867 attenuated the orexin-induced inward current (*P* < 0.01; Supplementary Figure 2), which was mimicked by selective OX2R agonist [Ala^11^, D-Leu^15^]-orexin B (Supplementary Figure 2) and nearly totally blocked by the potent dual orexin receptor antagonist TCS1102 (*P* < 0.01; Supplementary Figure 2). Moreover, OX1R or OX2R knockdown markedly lessened the orexin-induced excitation on GABAergic VP neurons (*P* < 0.01, respectively; Fig. 3a1–b2). These results suggest that both OX1R and OX2R are involved in the excitatory effect of orexin on VP neurons. Furthermore, KB-R7943, a selective blocker for Na^+^–Ca^2+^ exchanger (NCX), or CdCl_2_, a broad-spectrum blocker for voltage-gated Ca^2+^ channels, partly blocked the orexin-induced inward currents (*P* < 0.01, respectively; Fig. 3c, d and Supplementary Figure 3a). Combined application of KB-R7943 and CdCl_2_ nearly totally blocked the orexin-induced excitation (*P* < 0.001; Fig. 3c, d, and Supplementary Figure 3a), implying a dual downstream ionic mechanism involving both NCX and voltage-gated Ca^2+^ channels. Besides, orexin-A significantly enhanced the high-voltage-activated (HVA) (*P* < 0.01; Supplementary Figure 3b) rather than low-voltage-activated (LVA) calcium currents (*P* = 0.458; Supplementary Figure 3b), indicating an involvement of HVA but not LVA Ca^2+^ channels. Since HVA Ca^2+^ channels include L-, P/Q-, and N-subtypes, we further examined the HVA Ca^2+^ channel subtypes responsible for the excitation of orexin on VP neurons. As shown in Fig. 3e, f and Supplementary Figure 4b and c, the orexin-induced inward current was partially suppressed by nifedipine (selective blocker for L-type Ca^2+^ channel, *P* < 0.01) rather than ω-CTX GVIA (selective blocker for N-type Ca^2+^ channel, *P* = 0.483) or ω-CTX MVIIC (blocker for N- and P/Q- type Ca^2+^ channel, *P* = 0.687), suggesting that L-, rather than N- and P/Q-subtype of HVA Ca^2+^ channel contributes to the orexin-induced excitation. Furthermore, combined application of nifedipine and KB-R7943 totally blocked the excitation of orexin-A (*P* < 0.01; Fig. 3f and Supplementary Figure 4a). All these results strongly suggest that NCX together with L-type Ca^2+^ channel may be coupled to orexin receptors and mediate the excitatory effect of orexin on GABAergic VP neurons.

Given that orexin directly excites VP neurons, we microinjected orexin-A into the bilateral VPs to investigate the effect of orexin in VP on depressive-like behaviors. As shown in Fig. 3g, h, orexin significantly reduced the immobility time of rats in the FST (*P* < 0.05; Fig. 3g) and markedly increased the preference of rats for sucrose solution (*P* < 0.05; Fig. 3h). In addition, orexin did not influence the distance traveled (*P* = 0.619; Fig. 3i) and rearing numbers in the open field (*P* = 0.134; Fig. 3i). Thus, we speculate that the orexinergic afferent inputs may directly excite GABAergic VP neurons and subsequently prevent depressive-like behaviors.

### Endogenous orexin in VP does not affect novel social interaction

**Fig. 4:**
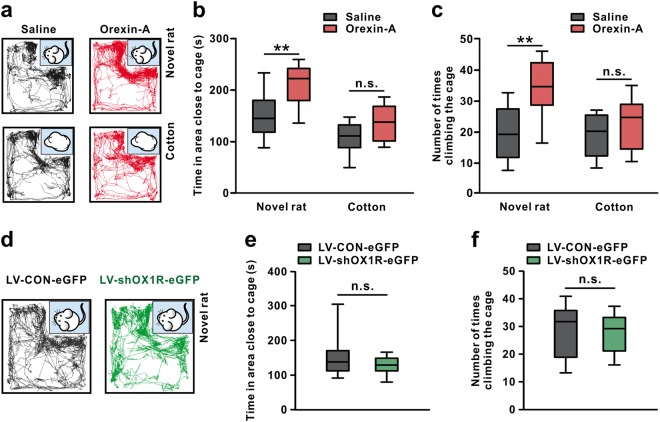
Endogenous orexin in VP does not affect novel social interaction. **a** Traces of rats microinjected with orexin-A or saline in the social interaction test. **b** Microinjection of orexin-A into bilateral VPs increased time that rats spent in investigating the area close to the caged novel rats (*n* = 10) rather than the caged cotton (*n* = 10). **c** Orexin-A-treated rats exhibited more times in climbing the cage when interacted with novel rat (*n* = 10) rather than cotton (*n* = 10). **d** Traces of rats treated with LV-shOX1R-eGFP or LV-CON-eGFP in the social interaction test. **e**, **f** Downregulation of OX1R did not influence the time rats spent in the area close to the caged novel rats (*n* = 9–10) (**e**) and the number of times rats climbing the cage (*n* = 9–10) (**f**) when interacting with novel rats. *n* = 10 for the LV-CON-eGFP group, *n* = 9 for the LV-shOX1R-eGFP group. Data are represented as median (horizontal bar) with 25th–75th (box) and 10th–90th (whiskers) percentiles; n.s. indicates not significant and **P < 0.01 by two-way ANOVA followed by Bonferroni-corrected *t* test (**b**, **c**) or by two tailed unpaired *t*-test (**e**, **f**)

Social avoidance is one of the crucial symptoms of depression. Therefore, we employed a social proximity test to assess the effect of orexin on social interaction between novel partners. As shown in Fig. 4, microinjection of orexin-A into bilateral VPs significantly increased the time of rats spent in the area close to a caged novel rat (*P* < 0.01; Fig. 4a, b) and the times of climbing the cage (*P* < 0.01; Fig. 4c). Nevertheless, neither the time spent close to the cotton (*P* = 0.084; Fig. 4a, b) nor the times of climbing the cage loaded with cotton (*P* = 0.280; Fig. 4c) was changed by orexin. These results, together with the above-mentioned result of open field test (Fig. 3i), indicate that exogenously injected orexin in VP may enhance the willingness of rats to interact with others, without change in general exploratory and locomotor activity.

To further determine the role of endogenous orexinergic inputs in VP in social interaction, we knocked down OX1R in VP by shRNA lentivirus. Unexpectedly, blockage of endogenous orexinergic inputs in VP did not change either the time rats spent in the area close to the caged novel rats (*P* = 0.325; Fig. 4d, e) or the times of climbing (*P* = 0.806; Fig. 4f). It suggests that endogenous orexin in VP may actually have no effect on novel social proximity, although exogenous orexin significantly promotes it.

### Endogenous orexin in VP alleviates social avoidance under acute psychosocial stress triggered by social rank

**Fig. 5:**
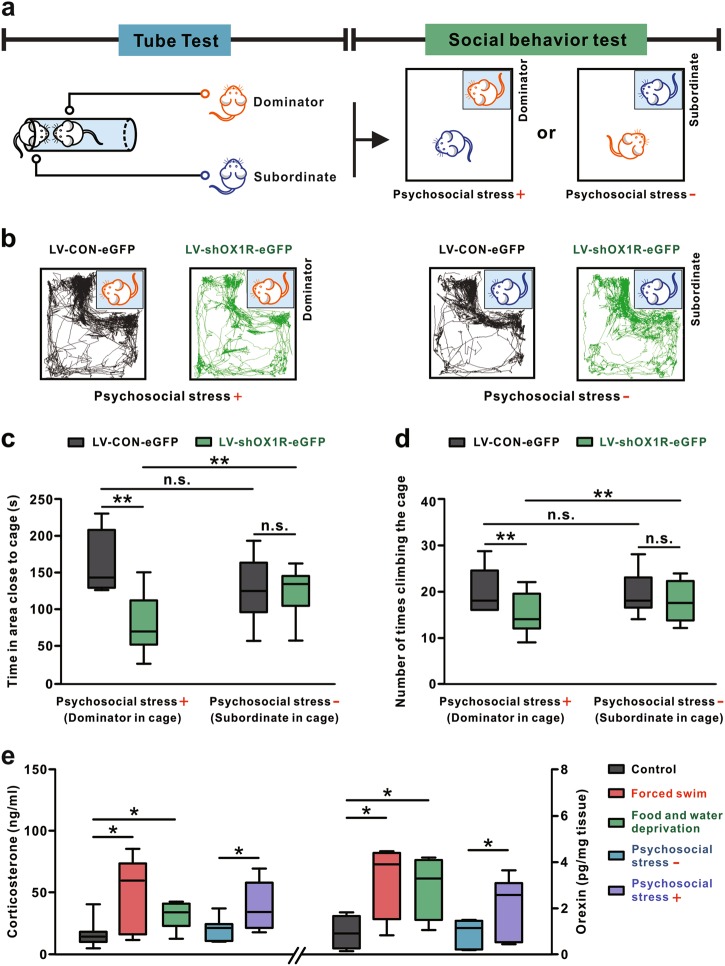
Endogenous orexin in VP alleviates social avoidance under acute psychosocial stress. **a** Scheme of experimental paradigm showing the social interaction tests, with or without psychosocial stress, between the pair of dominator and subordinate rat competed from an antecedent tube test. **b** Traces of rats treated with LV-shOX1R-eGFP or LV-CON-eGFP in the social interaction tests in the presence or absence of psychosocial stress. **c** Time that subordinate/dominator rats treated with LV-shOX1R-eGFP or LV-CON-eGFP spent in the area close to the caged dominator/subordinate (*n* = 9–10 per group). **d** Number of times of subordinate/dominator rats treated with LV-shOX1R-eGFP or LV-CON-eGFP climbing the cage in the presence and absence of psychosocial stress (*n* = 9–10 per group). *n* = 10 for the LV-CON-eGFP subordinate and LV-shOX1R-eGFP dominator group, and *n* = 9 for the LV-shOX1R-eGFP subordinate and LV-CON-eGFP dominator group. **e** ELISA analyses show serum corticosterone (*n* = 6–7 per group) and orexin-A level in VP (*n* = 12) under different stress and non-stress conditions. *n* = 6 for food/water deprivation group, and *n* = 7 for other groups. Data are represented as median (horizontal bar) with 25th–75th (box) and 10th–90th (whiskers) percentiles; n.s. indicates not significant and *P < 0.05, **P < 0.01, by two-way (**c**, **d**) or one-way (**e**) ANOVA followed by Bonferroni-corrected *t* test

The consistent effects of endogenous and exogenous orexin on forced swimming and sucrose preference, with the paradoxical effects of them on novel social interaction, impel us to reconsider the exact pathophysiological function of orexinergic modulation on VP in depression. Considering both FST and SPT are under acute stress (forced swimming and food/water deprivation) whereas novel SIT involves no apparent stress, we speculated that endogenous orexin in VP may be more critical for alleviation of depressive behaviors under stress than non-stress conditions. Given that social subordinance causes psychosocial stress and influences mental health [37, 38], we designed a novel paradigm by combination of SIT with the tube test (Fig. 5a), which is usually applied to assess social hierarchy [29, 39]. Pairs of dominator and subordinate rats competed from the tube test were used in the following SIT (Fig. 5a), during which the dominator exerted an acute psychosocial stress to the subordinate. Lentivirus was injected into VP 14 days before the tube test. Performances between OX1R knockdown rats, normal rats (LV-CON-eGFP), and OX1R knockdown rats vs normal rats in the tube test have no significant difference (*P* = 0.768; Supplementary Figure 5), indicating orexin in VP is not involved in the regulation of social hierarchy. Notably, we found that blockage of endogenous orexinergic inputs in VP by knockdown of OX1R significantly suppressed the social proximity of subordinates to caged dominators (*P* < 0.01; Fig. 5b–d), but had no influence on the social interaction between dominators and caged subordinates (time: *P* = 0.843; times of climbing: *P* = 0.344; Fig. 5b–d). The result implicates that endogenous orexin in VP may prevent social avoidance under psychosocial stress but not non-stress conditions.

Intriguingly, in normal rats (LV-CON-eGFP), no significant difference was found between social interaction of subordinates with caged dominators and that of dominators with caged subordinates (time: *P* = 0.073; times of climbing: *P* = 0.910, Fig. 5b–d). However, in OX1R knockdown rats (LV-shOX1R-eGFP), willingness of subordinates to interact with caged dominators was significantly lower than that of dominators to interact with caged subordinates (*P* < 0.01; Fig. 5b–d). These results indicate that psychosocial stress may induce social avoidance more easily in rats with knockdown of OX1R in VP. Therefore, we propose that the endogenous orexinergic system is likely to help to cope with acute stressful social experiences, while dysfunction of orexinergic inputs in VP may result in susceptibility to psychosocial stress and social avoidance in depression.

To confirm this hypothesis, we assessed serum corticosterone level, a marker for stress, and orexin concentration in VP, in rats facing the above different behavioral tasks. We found that serum corticosterone level was elevated with a significant increase in orexin level in VP 15 min after finishing exposure to acute stresses, including forced swimming, food/water deprivation and social rank stress (*P* < 0.05; Fig. 5e). However, both serum corticosterone level and orexin level in VP did not change in rats in non-stress situations, such as home cage and social interaction without psychosocial stress (orexin: *P* = 0.941; corticosterone: *P* = 0.519; Fig. 5e). Considering the stress-dependent elevation of orexin level in VP and the orexin-produced preventing effect in depressive paradigms under stress, we suggest that the direct orexinergic modulation on GABAergic VP neurons may prevent depressive-like behaviors especially by improving the capacity for resilience to stress.

## Discussion

Stress is one of the strongest risk factors for depression. Epidemiological studies show that up to 70–80% of major depressive episodes are preceded by stressful life events [40]. Thus, resilience, the capacity to bounce back from acute stress or more chronic forms of adversity, is critical for generation of adaptive physiological and psychological responses to stressors [4, 41] and protection against depressive symptoms [41, 42]. However, neural mechanisms underlying stress resilience are not clearly understood. Here, we demonstrate that orexin directly excites GABAergic VP neurons and consequently prevents depression by promoting stress resilience.

A growing body of experimental studies has indicated a close relationship between orexin and depression [13, 43, 44]. In clinic, suicidal patients with major depressive disorder exhibit reduced orexin levels in their cerebrospinal fluid [13, 45]. However, the neural substrates and mechanisms underlying role of orexin in depression still remain enigmatic. In this study, we reveal, for the first time, that orexin prevents depressive-like behaviors via VP. We show that hypothalamic orexinergic neurons project directly to VP and orexin depolarizes GABAergic VP neurons through OX1R and OX2R as well as their downstream NCX and L-type Ca^2+^ channel. Although VP lies within the basal ganglia, it constitutes a core component of the limbic loop rather than motor loop of basal ganglia [46]. Recently, VP has been revaluated as an important “limbic final common pathway” for mesocorticolimbic processing of reward [46, 47] and a hedonic hotspot [8, 46, 48, 49]. Notably, microinjection of orexin into the posterior VP enhances “liking” reactions to sucrose taste [48]. This orexin hedonic hotspot in VP is well positioned to mediate natural modulations of positive affect, and supposed to contribute to psychopathological conditions that may distort positive affect, such as depression [48]. Furthermore, besides anhedonia, other symptom domains in depression including behavioral despair and social withdraw are also encoded by discrete neuronal populations in VP that project to separate brain regions [12]. In the present study, we found that microinjection of orexin into VP not only enhanced sucrose preference, but also inhibited behavioral despair and increased proximity to novel social partners, whereas blockage of orexinergic inputs in VP induced the distinct core symptoms of depression. Since GABAergic neurons are the majority of neurons in VP and these principle neurons distributed across whole VP project to various brain regions related to depression, such as the lateral habenula and medial dorsal nuclei of thalamus [10, 50], our results suggest that orexinergic inputs widespread throughout the VP may prevent multiple depressive symptom domains via direct modulation on GABAergic neurons in both anterior and posterior VP, and VP may be an essential target of orexin and orexinergic system for protection against depression.

On the other hand, orexin and the central orexinergic system have emerged as a potential modulator in response to stress. It has been reported that orexinergic neurons are particularly active when rodents are facing acute psychological or physiological stressors, including electric foot shocks, restraint stress, and hypercapnia [51–54]. These are consistent with our findings that orexin level in VP elevates with an increase in serum corticosterone level when animals are exposed to forced swimming, food/water deprivation or social rank stress, rather than non-stress conditions. Furthermore, genetic knockdown or pharmacological blockade of orexin receptors in VP to block orexinergic inputs significantly enhanced depressive-like behaviors in the FST and SPT, and induced social avoidance of subordinates in the SIT to the caged dominators competed from the tube test, but did not affect social interaction between novel partners in non-stress situations. Therefore, these results substantially suggest that the deficiency in orexinergic system, especially orexinergic afferent inputs in VP, may result in vulnerability to acute stress and consequently manifest maladaptive behaviors and depression. Given that VP is also implicated in emotion and stress resilience [4–6], and orexinergic neurons are likely to promote adaptive behaviors in response to acute stress, such as improving avoidance performance in shock-associated contexts or increasing the time spent in safe and enclosed zones in an unfamiliar environment [51, 55], we propose that orexin and central orexinergic system may prevent depression by promoting stress resilience.

Effective strategies for prophylaxis and treatment of depression are still lacking. Stress, including psychosocial stress evoked by social hierarchy, greatly affects individual’s psychological health and results in depressive responses [38, 56]. Therefore, stress resilience and vulnerability is critical for the prevention and treatment of depression. In this study, we define an indispensable role for central orexinergic system in stress resilience and protection against depression. Orexin produces protective effect on various symptom domains of depression via direct modulation on GABAergic VP neurons. These findings not only reveal a neural circuit responsible for preventing depressive reactions to stress, but also provide a novel insight into the etiology, pathophysiology, prophylaxis, and treatment of depression.

## Electronic supplementary material

Supplementary information
