# Supplementary material for: Orexin prevents depressive-like behavior by promoting stress resilience
Source: Mol Psychiatry. 2018 Aug 7;24(2):282–93. doi: 10.1038/s41380-018-0127-0 (PMC6755988; doi:10.1038/s41380-018-0127-0)
Supplement: Supplementary file 1 — Supplementary information [file 41380_2018_127_MOESM1_ESM.pdf]

Supplementary Information for

Orexin prevents depressive-like behavior by promoting resilience to acute stress

Miao-Jin Ji†, Xiao-Yang Zhang†, Zi Chen, Jian-Jun Wang*, Jing-Ning Zhu*

Correspondence to: jnzhu@nju.edu.cn **(J.-N.Z.);** jjwang@nju.edu.cn **(J.-J.W.).**

This PDF file includes:

Supplementary Materials and Methods

Supplementary Figures 1 to 5

SUPPLEMENTARY MATERIALS AND METHODS

Animals

Adult Sprague-Dawley rats (8 weeks, 200-250 g; Experimental Animal Center of Nanjing Medical University, Nanjing, China) were housed under a 12-hour light/dark cycle, with free access to food and water except for a specific description. All experimental procedures were conducted in accordance with the U.S. National Institutes of Health Guide for the Care and Use of Laboratory Animals (NIH Publication 85-23, revised 2011) and approved by the Experimental Animal Care and Use Committee of Nanjing University.

Lentivirus and drug microinjection

Lentivirus production. To downregulate the expression of individual orexin receptor subtype in the VP, shRNA lentiviruses targeting rat OX1R and OX2R mRNAs were constructed. The sequences used against rat orexin receptor subtypes were as follows: *ox1r*, TCACCGAATCATGGCTCTT; *ox2r*, GGACGCACAAGTACAGAGA. A scrambled shRNA (TTCTCCGAACGTGTCACGT) was used as a control. The shRNA sequences were cloned into the lentiviral vector GV248 (pFU-GW-007-hU6-Ubiquitin-EGFP-IRES-puromycin; GeneChem, Shanghai, China). The 293T packaging cells were cotransfected with GV248 carrying shRNA, pHelper 1.0 and

pHelper 2.0 plasmids using Lipofectamine 2000 (Invitrogen, Carlsbad, CA) to amplify and package the recombinant lentivirus (LV-shOX1R-eGFP, LV-shOX2R-eGFP and LV-CON-eGFP). After 24 h incubation, medium was replaced with fresh DMEM and culture was continued for 48 h at 37˚C. The viral supernatant was then collected, filtered, and concentrated. Titers of concentrated viral particles were 1×10^9 TU/ml.

Stereotaxic surgery. Adult male rats were anesthetized using sodium pentobarbital (40 mg/kg, i.p.) and mounted on a stereotaxic frame (1404, David Kopf Instruments, Tujunga, CA) for stereotactic brain surgery under aseptic conditions. Throughout the surgery, body temperature was monitored by a rectal thermistor probe and maintained at 36-38°C on a heating pad. The scalp was incised to expose the skull. Small holes were drilled in the skull above the targeted coordinates according to the rat brain atlas of Paxinos and Watson[1], and the dura was gently broken to allow uninterrupted passage of Hamilton syringes or microinjection cannulae.

Intra-VP lentivirus microinjection. The concentrated lentivirus was delivered into the VP bilaterally (A 0.0 - -0.3, L 2.5, and H 7.6) using a 1 μl Hamilton syringe with a thin 25-gauge metal needle, and the injection was driven by an infusion pump (KDS100, KD Scientific, Holliston, MA; the injection volume and flow rate 1 μl at 0.1 μl/min). After the injection, the needle was left in place for 10 additional minutes and then slowly withdrawn. The rats treated with lentivirus were caged individually and allowed to recover for 14 days before further behavioral studies. eGFP positivity in the VP was

determined by a Leica STP 8000 confocal microscope (Leica, Wetzlar, Germany). The downregulation of expression of orexin receptor subtypes in the VP was assessed by quantitative real time RT-PCR and western blot.

Intra-VP drug microinjection. For pharmacological blockage of orexin receptors in VP, two stainless-steel guide tubes (length 11 mm, o.d. 0.8 mm, i.d. 0.5 mm) for the microinjection cannulae were implanted into bilateral VPs of each rat. The lower ends of guide tubes were positioned 2.0 mm above the VP (A 0.0 - -0.3, L 2.5, and H 7.6). After the implantation, animals were caged individually and allowed to recover for at least 3 days. During the behavioral testing sessions, two stainless-steel injection tubes (length 13mm, o.d. 0.5 mm, i.d. 0.3 mm) were inserted to protrude 2 mm beyond the tip of the guide tubes and just above the VP (to minimize lesioning the nuclei) for microinjection of orexin A (1 μM, Tocris, Bristol, UK), TCS1102 (20 nM, Tocris), and saline (0.9 % NaCl) using Hamilton syringes (0.5 μl each side, lasting 2 min). The effective extent of the drug diffusion in the present study was estimated by using extracellular electrophysiological recording units 0.5-2.0 mm away from the injection site according to our previous reports[2,3] and restricted in the VPs.

Behavioral assessments

Forced swim test. The forced swim test took place in a vertical glass cylinder (50 cm in height × 20 cm in diameter) containing 35 cm of freshwater at 25 ± 2 °C. In the

first day, the rats were forced to swim for 10 min and thereafter dried with heater. After 24 h, rats were re-exposed to forced swimming for 10 min and behavior was videotaped. The total time spent immobile were measured by video analysis (TopView Animal Behavior Analyzing System; CleverSys Inc, Reston, VA).

Sucrose preference test. Rats were habituated to one bottle of water and one bottle of 1% sucrose in home cages for 1 week. Sucrose consumption was then measured for one-hour preceded by a 20 hours food and water deprivation. The amount of fluid intake was measured by weighing the bottles before and after the one-hour test. The preference score was calculated as sucrose intake relative to total fluid intake. The sucrose preference baseline was averaged from two baseline fluid intake tests performed before rat grouped and separated by at least 5 days. Sucrose preference tests were conducted immediately after drug microinjection or weekly after lentivirus infusion.

Open field test. Rats were tested in a sound-isolated, dimly illuminated room (20 lux) in an open-field box (50 cm × 50 cm). Rats were allowed to explore the box freely and their behavior was recorded for 10 min. The number of times rats rearing and the total distance they travelled were calculated (TopView Animal Behavior Analyzing System).

Novel social proximity test. In each test, two rats that met for the first time were randomly selected from separate cages. The tested rat was placed in the center of a

plastic open field apparatus (50 × 50 cm) simultaneously with the other novel partner or a cloud of cotton which was put in an iron cage (16 × 22 cm) in one corner of the arena. Time of head approaching and staying close to the cage (≤ 5 cm) and the times of forepaws climbing the cage in 10 minutes were recorded and calculated (TopView Animal Behavior Analyzing System).

Social interaction test under psychosocial stress. A pair of rats in similar weight were simultaneously loaded into the opposite ends of a plexiglass pipe tube (length 60 cm, o.d. 72 mm, i.d. 68 mm) and held until both rats strived to move forward. A trial will last until one of the rats is pushed out, or for a maximum of 5 minutes. The winner for 3 repeated trials was considered as a dominator and the losing rat as subordinate. The pair of rats was conducted to test social proximity 15 min after the tube test, in which the caged dominator was introduced as a psychosocial stressor for subordinate.

After behavioral tests, the animals were deeply anaesthetized and the microinjection sites were histologically identified as we previously reports.[3,4] Data from rats in which the injection sites were deviated from the VP were excluded from further analysis.

Quantitative real time RT-PCR

In this experiment, three independent groups of RNA pools each from five adult

animals were used as biological replicates. VP tissue punches were collected from coronal brain slices according to the rat brain atlas of Paxinos and Watson[1]. RNA extraction was carried using TriZol reagent (Invitrogen, Carlsbad, CA) according to the manufacturer's instructions. A 1 μg aliquot of total RNA was used for the first-strand cDNA synthesis according to the protocol of Prime Script RT reagent kit (Takara, Japan). Real-time PCR was then performed using Fast Start Universal SYBR Green Master (Roche Diagnostics, Indianapolis, IN) in a 20 μL of reaction mixture containing 10 μL of 2 × master mix, 2 μL of cDNA, 2 μL of each primer (5 μM) and 4 μL of distilled water. The reaction was carried out in the ABI Prism 7500 System (Applied Biosystems, Foster City, CA) using the following parameters: 95°C for 3 min to activate the hot-start iTaq DNA polymerase, followed by 40 cycles at 95°C for 15 s, 60°C for 25 s and 72°C for 1 s. The PCR program was completed by a melting temperature analysis. For quantification, the quantity of the target gene was expressed relative to the amount of the reference gene (*gapdh*) to obtain a normalized target expression value. For negative controls, cDNA was replaced with water.

Primer sequences were summarized as follows. *ox1r* (NM_013064) forward: 5'-GGC TGG TGT ACG CCA ACA G-3' and reverse: 5'-TTG AAC TGC TCC CGA AAT TTG-3'; *ox2r* (NM_013074) forward: 5'-AGA TCC GAG CAC GAA GGA AA-3' and reverse: 5'-ATA GCA AAT TGC AAA GAC CAG AAG T-3'. *gapdh* (NM_017008) forward: 5'-GGT GCT GAG TAT GTC GTG GAG TCT AC-3' and reverse: 5'-CAT GTA GGC CAT GAG GTC CAC CAC C-3'.

Western blot

For western blot analysis, tissue lysate from dissected rat VP was used. VP tissue was homogenized in 200 μl of lysis buffer (1% Nonidet P-40, 20 mM Tris, pH 8.0, 137 mM NaCl, 10% glycerol, 1 mM PMSF, sodium butyrate 1 mM, and protease inhibitors) at 4°C. After removal of cellular debris by centrifugation, the supernatant was collected, and protein levels in the lysates were measured by the Bradford assay (Bio-Rad, Hercules, CA). 50 μg of each sample was boiled in the presence of sample buffer for 5 min before separation on 10% SDS polyacrylamide gel, and proteins were transferred to nitrocellulose membranes. The immunoblots were blocked with 5% nonfat dry milk dissolved in PBS for 60 min. The membranes were then incubated overnight at 4°C with primary antibody: rabbit anti-OX1R (1:250; Abcam, Cambridge, MA; Cat# ab68718, RRID:AB_1269637), goat anti-OX2R (1:50; Santa Cruz, Santa Cruz, CA; Cat# sc-14394, RRID:AB_2117941), rabbit anti-GADPH (1:2500; Abcam; Cat# ab37168, RRID:AB_732652), rabbit anti-β-actin (1:2500; Abcam; Cat# ab8227, RRID:AB_2305186). Primary antibody incubation was followed by three washes (5 min, rocking, room temperature) in PBST (PBS containing 0.2% Tween 20) before incubation with the secondary antibody (Far red light/infrared testing western blotting fluorescent resistance goat anti-rabbit IgG; Jackson ImmunoResearch, West Grove, PA), three washes. Protein expression was detected with Infrared Imaging Systems (LI-COR Inc, Lincoln, NE). GAPDH and β-actin were used as loading control. The optical

densities of protein bands were quantitatively analyzed with Quantity One software (Bio-Rad).

Retrograde tracing with Fluoro-Gold

For retrograde tracing experiments, stereotaxic surgery was conducted on adult rats as described above.[4] A glass micropipette (WPI, Sarasota, FL, USA) with inner tip diameter of 10–15 μm was filled with 2% Fluoro-Gold (FG; Fluorochrome, Denver, CO) in 0.01 M phosphate buffered saline (PBS, pH 7.4) and then lowered slowly into the VP at the coordinates A 0.0 to -0.3, L 2.5, and H 7.6 according to the rat brain atlas of Paxinos and Watson[1]. Iontophoresis were made under 5 μA positive alternating current (7 s on/7 s off) for 30 min. Once completed, the micropipette was left in place for 10 min before removal. During micropipette withdrawal, the current was reversed to minimize tracer leakage through the injection tract. The incision was closed, and the animals were placed into separate cages to survive for 14 days before perfusions. Experimental conditions were repeated three times to account for technical and biological variation.

Immunohistochemistry

Adult rats were given an overdose of sodium pentobarbital and perfused transcardially with 100 ml of saline, followed by 450-500 ml of 4% sodium phosphate-

buffered paraformaldehyde. Brains were post-fixed in the same fixative for 12 h at 4 °C, then cryoprotected with 30% sucrose for 48 h. Frozen coronal sections (15 μm thick) were obtained by using a freezing microtome (CM3050S, Leica) and mounted on gelatin-coated slides. The slices were rinsed with PBS containing 0.1 % Triton X-100 and then incubated in 10 % normal bovine serum in PBS containing 0.1 % Triton X-100 for 30 min. Sections were incubated overnight at 4 °C with primary antibodies: mouse anti-orexin A monoclonal antibody (1:200; R&D, Minneapolis, MN; Cat# MAB763, RRID:AB_2117627), rabbit anti Fluoro-gold antibody (1:2000; Millipore, Boston, MA; Cat# AB153, RRID:AB_90738), rabbit anti OX1R antibody (1:100; Abcam; Cat# ab68718, RRID: AB_1269637), goat anti OX2R antibody (1:100; Everest, Waltham, MA; Cat# EB08124, RRID:AB_2117786), mouse anti GABA (1:1000; Sigma, St. Louis, MO; Cat# A0310, RRID: AB_476667). After a complete wash in PBS, the sections for single and double immunostaining were incubated in the related Alexa 488-, Alexa 568-, Alexa 405, streptavidin Alexa 594-conjugated secondary antibodies (1:2,000; Life technologies, Carlsbad, CA) for 2 h at room temperature in the dark. The slides were washed and mounted in Fluoromount-G mounting medium (Southern Biotech, Birmingham, AL). Incubations replacing the primary antiserum with control immunoglobulins and/or omitting the primary antiserum were used as negative controls. All micrographs were taken with a Leica STP 8000 confocal microscope, equipped with Plan-Apochromat × 60/1.42 NA oil, ×40/0.9 NA dry, ×20/0.75 NA dry, and ×10/0.4 NA dry objective lenses. Digital images from the microscope were recorded with Leica Application Suite (v2.5.0 R1) and image processing was done with Image Pro Plus (6.0).

Experimental conditions were repeated at least three times to account for technical and biological variation.

For quantitation of density of orexinergic fibers localized in VP, 25 μm coronal sections were obtained across the antero-posterior axis (+0.48 to -0.6 mm relative to Bregma) of the brain and imaged on a Leica STP 8000 confocal microscope. For each rat, orexinergic fiber density, being assessed by the surface area (μm^2) occupied by orexin-A immunoreactivity per mm^2 of the VP, was quantitated every second section with Image Pro Plus (6.0). Data are presented as an average density of orexinergic fibers in the anterior (+0.48 to 0 mm from Bregma) and posterior (0 to -0.6 mm from Bregma) region of the VP.

Whole-cell patch clamp recordings on brain slices

Thirty-seven adult rats of either sex were decapitated under sodium pentobarbital anaesthesia. After decapitation under sodium pentobarbital (40 mg/kg) anesthesia, the coronal slices (300 μM in thickness) containing VP were obtained according to the rat brain atlas[1]. The slices were incubated in oxygenated artificial cerebrospinal fluid (ACSF, composition in mM: 124 NaCl, 2.5 KCl, 1.25 NaH_2PO_4, 1.3 $MgSO_4$, 26 $NaHCO_3$, 2 $CaCl_2$ and 10 D-glucose) at 35 ± 0.5 °C for at least 1 h. Then the slices were maintained at room temperature for about 20 min before recordings, and were transferred to a submerged chamber during recording sessions and continuously

perfused with oxygenated ACSF at a rate of 2 ml/min maintained at 32 ± 0.5 °C.

Whole-cell patch clamp recordings were performed on VP neurons with borosilicate glass pipettes (3-5 MΩ) filled with (in mM): 140 K-methylsulfate, 7 KCl, 2 $MgCl_2$, 10 HEPES, 0.1 EGTA, 4 Na_2-ATP, 0.4 GTP-Tris, and 4% biocytin (Sigma-Aldrich, St. Louis, MO), pH 7.25. During recording sessions, VP neurons were visualized with an Olympus BX51WI microscope (Olympus, Tokyo, Japan). For recording VP neurons with knockdown of OX1R or OX2R, a xenon light source and an MTI-Dage CCD camera (IR-1000, Meyer Instruments, Houston, TX) were used to detect the bright fluorescence signal of eGFP-positive neurons. Patch clamp recordings were acquired with an Axopatch-200B amplifier (Axon Instruments, Foster City, CA) and the signals were fed into a computer through a Digidata-1550 interface (Axon Instruments) for data capture and analysis (pClamp 10.0, Axon Instrument). Recordings of whole-cell currents were low-pass filtered at 2 kHz and digitized at 10 kHz and recordings of membrane potentials were low-pass filtered at 5 kHz and digitized at 20 kHz. Neurons were held at a membrane potential of -60 mV and characterized by injection of rectangular voltage pulse (5 mV, 50 ms) to monitor the whole-cell membrane capacitance, series resistance and membrane resistance. Neurons were excluded from the study if the series resistance was not stable or exceeded 25 MΩ. To record voltage-gated Ca^{2+} current, depolarizing voltage steps were employed under voltage-clamp mode. Low-voltage-activated (LVA) Ca^{2+} current was evoked at -40 mV (200 ms) from a holding potential of -90 mV, and high-voltage-activated (HVA) Ca^{2+}

current was evoked at 0 mV (200 ms) from a holding potential of -60 mV.

We bathed the slices with orexin A (300 nM, Tocris, Bristol, UK) to stimulate the recorded neurons. TTX (0.3 μM, Alomone Labs, Jerusalem, Israel) was used to confirm and isolate the postsynaptic response of the recorded neuron to orexin A. A selective OX1R antagonist, SB334867 (3 μM, Tocris), a dual orexin receptor antagonist, TCS1102 (20 nM, Tocris), as well as a selective agonist for the OX2R, [Ala^{11}, D-Leu^{15}]-orexin B (0.3 and 1 μM, Tocris) were applied to assess the underlying postsynaptic receptor mechanisms. KB-R7943 (30 μM, Tocris) and $CdCl_2$ (Sigma) were applied to block the Na^+-Ca^{2+} exchanger (NCX) and Ca^{2+} channels, respectively. Furthermore, selective blockers for L-type and N-type voltage-gated calcium channels (VGCCs), nifedipine (10 μM, Sigma) and ω-CTX GVIA (1 μM, Bachem, Bubendorf, Switzerland), as well as a N- and P/Q- type VGCCs blocker, ω-CTX MVIIC (1 μM, Bachem) were used to discriminate the specific type of VGCCs coupled to orexin receptors. Besides, the selective Na^+ channel blocker tetrodotoxin (TTX, 0.3 μM), the relatively selective A-type K^+ channel blocker 4-aminopyridine (4-AP, Sigma) at a low concentration (500 μM), as well as the K^+ channel blocker tetraethylammonium chloride (TEA, 20 mM; Sigma) and Cs^+ (5 mM, Sigma), were employed to eliminate Na^+ and K^+ component in isolating Ca^{2+} current. Moreover, Cs^+-based internal pipette solution was used to block K^+ channels from the inside of membrane. Cs^{2+}-based pipette solution was composed of the following (in mM): 100 CsCl, 20 TEACl, 5 $MgCl_2$, 2 BAPTA, 10 HEPES, 5 Na_2ATP, 0.5 Na_3GTP, and CsOH/HCl for pH 7.3. Sucrose was added to bring the

osmolality to 305 mOsm.

Sample processing for ELISA

Fifteen minutes after behavioral test, the rats were anesthetized with isoflurane then drawn blood from eyeball and decapitated. Blood was collected in serum tubes and brain tissue sample was dissected similar to those described in our previous publication.[5,6] For the blood sample, blood was allowed to coagulate at room temperature in serum tubes for 1 hour then centrifuged 3000 rpm for 10 min. Serum was aliquoted and stored at -80 °C. For the brain tissue sample collected from the VP, tissue was weighed and immersed in 0.5 M acetic acid and then boiled for 10 min. The VP samples were centrifuged for 30 s at 5000 rpm and the supernatants were air dried under a hood overnight, and the dried samples were subsequently stored at -80 °C. Corticosterone kit (KGE009; R&D Systems Inc., Minneapolis, MN, USA) and orexin-A kit (FEK-003-30; Phoenix Pharmaceuticals, Dayton, OH, USA) were used for the ELISA testing. Upon completion of the assays, the fluorescence intensities of the 96 well microplates were read by an assay reader (Tecan, Männedorf, Switzerland).

Experimental design and statistical analysis

All experiments and data analysis were performed blind to the conditions of the experiments. In behavioral experiments, the animals were randomly grouped by

different treatments. Sample sizes were based on previous experiments.[3,4,7,8] All data were analyzed with SPSS 17.0 and presented as median (horizontal bar) with 25th-75th (box) and 10th-90th (whiskers) percentiles. Data were tested for normal distribution and homogeneity of variance. Two-tailed unpaired and paired Student's t test, one-way, two-way and repeated measures two-way analysis of variance (ANOVA), and post hoc Bonferroni-corrected t test was employed for statistical analysis. P-values of < 0.05 were considered to be significant.

REFERENCES

1. Paxinos G, Watson C. *The Rat Brain in Stereotaxic Coordinates (seventh ed.)*. Academic Press, San Diego, 2014.
2. Song YN, Li HZ, Zhu JN, Guo CL, Wang JJ. Histamine improves rat rota-rod and balance beam performances through H_2 receptors in the cerebellar interpositus nucleus. *Neuroscience* 2006; **140**: 33–43.
3. Zhang J, Li B, Yu L, He YC, Li HZ, Zhu JN *et al*. A role for orexin in central vestibular motor control. *Neuron* 2011; **69**: 793–804.
4. Wang Y, Chen ZP, Zhuang QX, Zhang XY, Li HZ, Wang JJ *et al*. Role of corticotropin-releasing factor in cerebellar motor control and ataxia. *Curr Biol* 2017; **27**: 2661–2669.

5. Zhang XY, Yu L, Zhuang QX, Peng SY, Zhu JN, Wang JJ. Postsynaptic mechanisms underlying the excitatory action of histamine on medial vestibular nucleus neurons in rats. *Br J Pharmacol* 2013; **170**: 156–169.

6. Peng SY, Zhuang QX, Zhang YX, Zhang XY, Wang JJ, Zhu JN. Excitatory effect of norepinephrine on neurons in the inferior vestibular nucleus and the underlying receptor mechanism. *J Neurosci Res* 2016; **94**: 736–748.

7. Borowsky B, Durkin MM, Ogozalek K, Marzabadi MR, DeLeon J, Lagu B. Antidepressant, anxiolytic and anorectic effects of a melanin-concentrating hormone-1 receptor antagonist. *Nat Med* 2002; **8**: 825–830.

8. Andrus BM, Blizinsky K, Vedell PT, Dennis K, Shukla PK, Schaffer DJ *et al*. Gene expression patterns in the hippocampus and amygdala of endogenous depression and chronic stress models. *Mol Psychiatry* 2012; **17**: 49–61.

SUPPLEMENTARY FIGURES

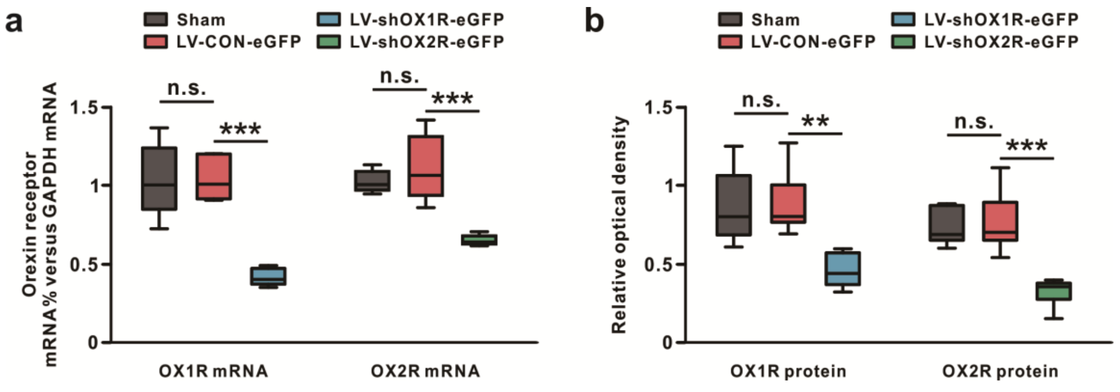

Supplementary Figure 1. Identification of the downregulation efficiency of *ox1r* and *ox2r* mRNAs and proteins by qPCR and western blot. (a) The expression level of *ox1r* and *ox2r* mRNAs relative to *gapdh* mRNAs in VP. LV-shOX1R-eGFP significantly decreased *ox1r* mRNA in VP (n = 5), so did LV-shOX2R-eGFP (n = 5). **(b)** The relative optical density of *ox1r* and *ox2r* proteins in VP. LV-shOX1R-eGFP significantly decreased *ox1r* proteins in VP (n = 6), so did LV-shOX2R-eGFP (n = 6). Data are represented as median (horizontal bar) with 25th-75th (box) and 10th-90th (whiskers) percentiles; n.s. indicates not significant and **P < 0.01, ***P < 0.001 by one-way ANOVA followed by Bonferroni-corrected t test.

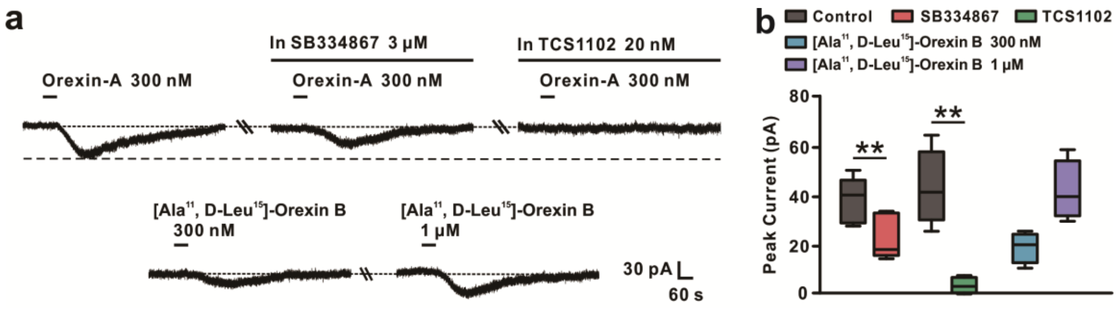

Supplementary Figure 2. OX1R and OX2R co-mediate the orexin-induced excitatory effect on VP neurons. (**a**) Orexin-A-induced inward current was partially blocked by SB334867 (highly selective OX1R antagonist, n = 5), and totally blocked by TCS1102 (highly selective dual OX1R and OX2R antagonist, n = 5), and mimicked by [Ala^{11}, D-Leu^{15}]-orexin B (highly selective OX2R agonist, n = 5). (**b**) Group data of the tested VP neurons. Data are represented as median (horizontal bar) with 25th-75th (box) and 10th-90th (whiskers) percentiles; **P < 0.01, by two tailed paired t-test.

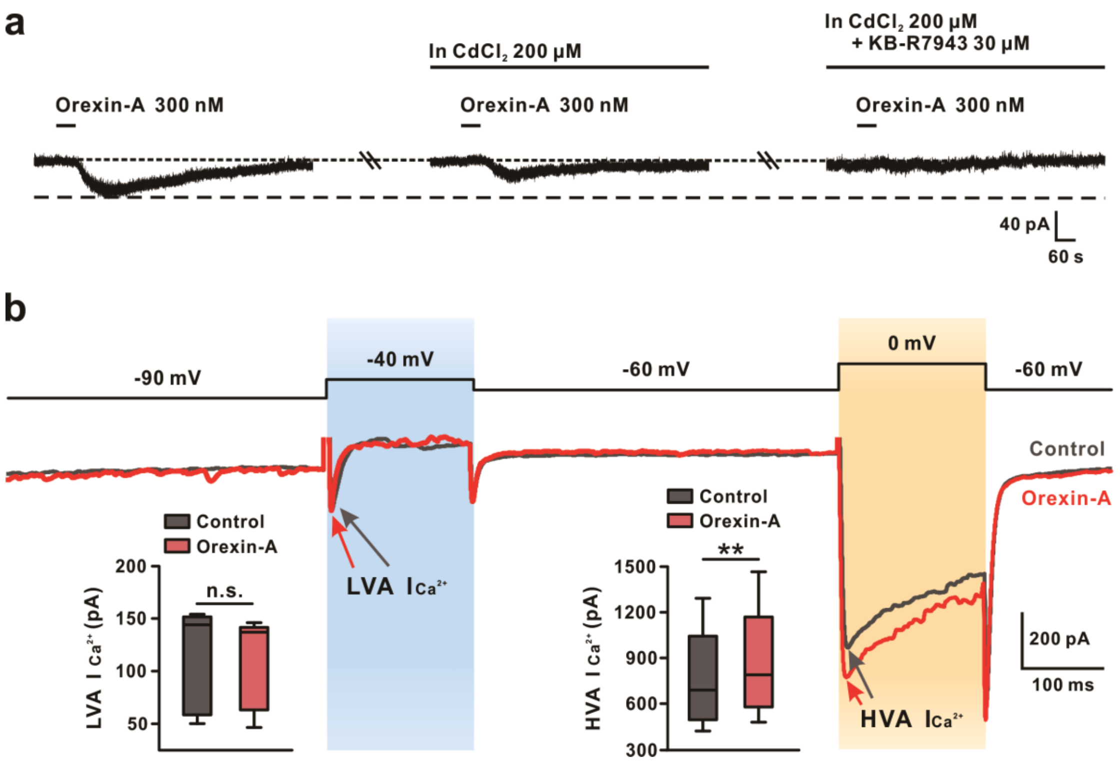

Supplementary Figure 3. Orexin A excites VP neurons via activating NCX and HVA Ca^{2+} channel. Patch clamp recordings on VP neurons showed that **(a)** $CdCl_2$, a broad-spectrum inhibitor of HVA Ca^{2+} channels, partly blocked the orexin-A elicited inward current, and combined application of $CdCl_2$ and a selective NCX blocker KB-R7943 totally blocked the current. **(b)** Traces of small LVA Ca^{2+} currents and large HVA Ca2+ currents evoked by depolarizations of −90 to −40 mV (200 ms) and −60 to 0 mV (200 ms) respectively, before and after bath application of orexin-A. Orexin-A enhanced HVA (n = 5) rather than LVA Ca^{2+} currents (n = 5). Data are represented as median (horizontal bar) with 25th-75th (box) and 10th-90th (whiskers) percentiles; n.s. indicates not significant and **$P < 0.01$ by two-tailed paired t test.

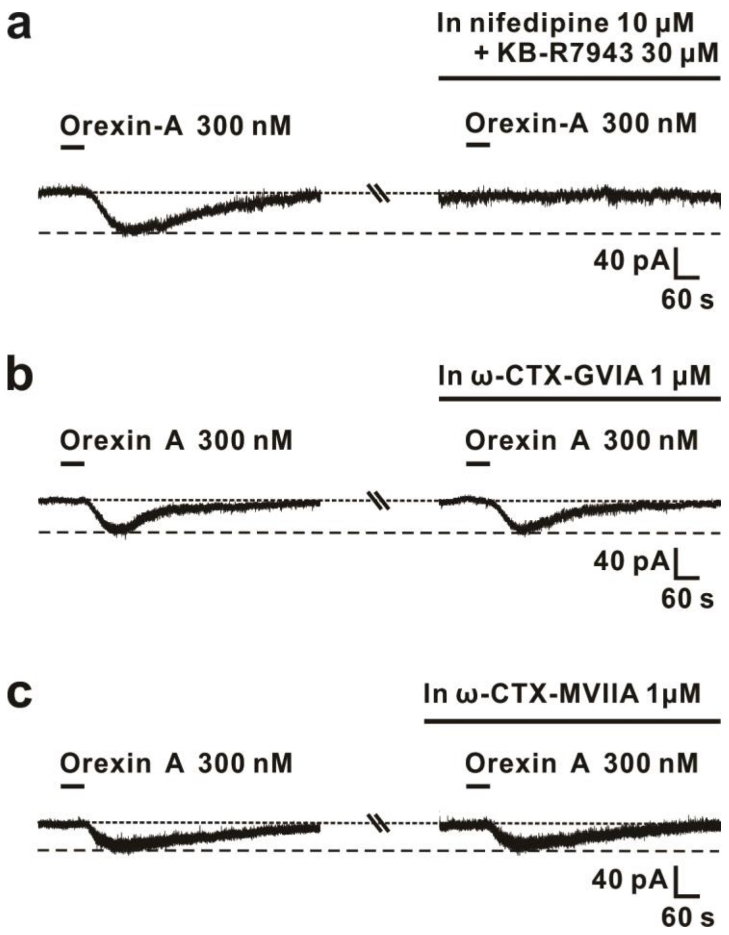

Supplementary Figure 4. Orexin A excites VP neurons via activating NCX and L-type Ca^{2+} channel. Patch clamp recordings on VP neurons showed that **(a)** combined application of a selective L-type Ca^{2+} channel blocker nifedipine and KB-R7943 totally blocked the orexin-A elicited inward current; **(b, c)** the inward current induced by orexin-A was affected by neither a selective N-type HVA Ca^{2+} channel blocker ω-CTX GVIA **(b)** nor a nonselective N/P/Q-type HVA Ca^{2+} channel blocker ω-CTX MVIIC **(c)**.

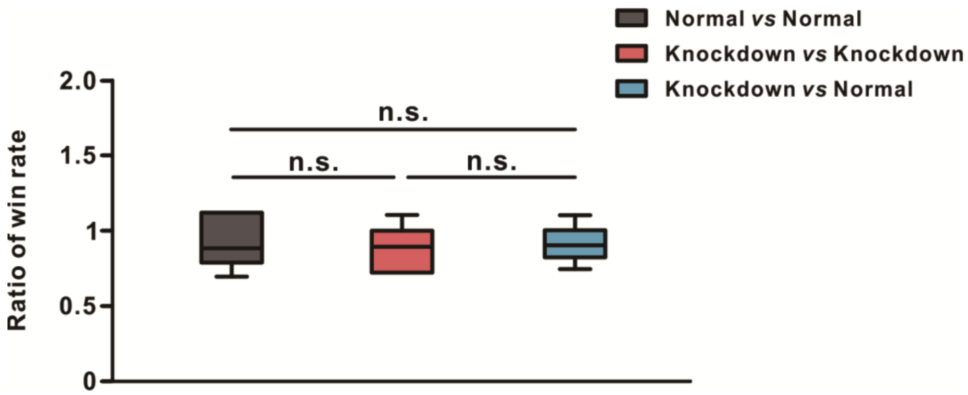

Supplementary Figure 5. Knockdown of OX1R has no effects on the performance of rats in tube test. Performances between normal rats (n = 17 pairs), OX1R knockdown rats (n = 19 pairs), and OX1R knockdown rats vs normal rats (n = 21 pairs) in the tube test have no significant difference, indicating orexin in VP is not involved in the regulation of social hierarchy. Data are represented as median (horizontal bar) with 25th-75th (box) and 10th-90th (whiskers) percentiles; n.s. indicates not significant by one-way ANOVA followed by Bonferroni-corrected t test.
